# Does Repeated Methamphetamine Exposure at Different Regimens Cause Parkinsonian-Like Behavior in Rats?

**Published:** 2018

**Authors:** Neda Valian, Abolhassan Ahmadiani, Leila Dargahi

**Affiliations:** a *Neuroscience Research Center, Shahid Beheshti University of Medical Sciences, Tehran, Iran. *; b *Neurobiology Research Center, Shahid Beheshti University of Medical Sciences, Tehran, Iran.*

**Keywords:** Methamphetamine, Motor impairments, Parkinson’s disease, Narrow beam test, Pole test

## Abstract

Methamphetamine (MA), a highly addictive psychostimulant, produces long-lasting neurotoxic effects well proven in nigrostriatal dopaminergic neurons. Considering the similarities between pathological profile of MA neurotoxicity and Parkinsonʹs disease (PD), some reports show that previous MA abusers will be at greater risk of PD-like motor deficits. To answer the question if repeated MA exposure causes parkinsonian-like behavior in rats, we used three regimens of MA administration and assessed the motor performance parameters immediately and over a long period after MA discontinuation. Male Wistar rats in two experimental groups were treated with escalating paradigms consisting of twice daily intraperitoneal injection of either 1-7 mg/kg or 1-14 mg/kg of MA over 14 days. The third group received twice-daily doses of 15 mg/kg of MA every other day for total number of 7 days. At the 1^st^, 7^th^, 14^th^, 21^st^, 28^th^, and 60^th^ days after last injections, motor activities were evaluated using narrow beam, pole, and rotarod tests. Locomotor activity was also evaluated using open field test. Repeated-measures ANOVA indicated that over the two months period following MA exposure, drug-treated rats perform beam, pole, and rotarod tests equally well as their corresponding vehicle-treated controls. Comparison of the locomotor activity didnʹt show significant differences between groups. These data indicated that MA at these regimens does not cause PD-related motor deficits in rats. Since MA doses, exposure duration, and dosing intervals have been shown to affect MA-induced dopaminergic toxicity, it can be concluded that none of these regimens; are strong enough to produce measurable behavioral motor deficits in rat.

## Introduction

Methamphetamine (MA) is a psychostimulant drug with high potential for abuse and addiction. It increases the release of monoamines, especially dopamine, from presynaptic terminals in the brain. MA can produce long-lasting deficits in dopaminergic cell bodies and terminals in the long-term use (1, 2). Among the different dopaminergic pathways in the brain, nigrostriatal neurons are more sensitive to MA-induced neurotoxicity, whereas mesolimbic and mesocortical pathways are minimally affected (3, 4). There is an evidence that repeated administration of MA reduces the striatal markers of dopaminergic nerve terminals including dopamine level, its metabolites, biosynthetic enzymes, receptors, and transporters in rodents (5), nonhuman primates (6), and human (7). Furthermore, MA induces neuronal apoptotic death and reduction in dopaminergic neuron cell bodies of substantia nigra (8-10). 

Given that about 70% of dopaminergic neurons in the central nervous system are located in substantia nigra (11), it is reasonable to suppose that long-term MA abuse causes parkinsonian-like motor deficits due to reduction in nigrostriatal neurons and terminals. In line with this, imaging studies have shown reductions in dopamine transporter (DAT) binding densities in MA abusers which persist following MA withdrawal for at least between 11 months (12), and 3 years (13, 14) that are associated with reduced motor skills (12). Postmortem studies also reported MA-induced dopaminergic neurotoxicity in the caudate and putamen (7, 13 and 15), which are paralleled neurochemical changes in Parkinsonʹs disease (PD) patients (13, 16). Moreover, using transcranial sonography, Todd *et al.* found an abnormal morphology of substantia nigra in individuals with a history of MA abuse, associated with reduced dopamine uptake in the striatum and increased risk for development of PD later in life (17). In addition to these pathological researches, several retrospective and prospective epidemiological studies showed increased risk of developing PD in humans who abuse MA compared to other drug abusers or non-abusers (18-20). 

Therefore, to establish a rat model of long-lasting motor deficits in former MA abusers, we evaluated whether repeated administration of MA can induce parkinsonian-like motor deficits in rats immediately and over a long period after MA exposure.

## Experimental

*Animals*

The subjects were adult male Wistar rats, weighting 220–250 g, obtained from our breeding colony (Neuroscience Research Center). Animals were housed as five in a cage with food and water available ad libitum, under a standard 12h-light/12h-dark cycle and temperature of 23 ± 2 ºC. The rats were allowed 5–6 days of habituation to the animal colony. All the experiments followed the National Institutes of Health guide for the care and use of laboratory animals (NIH Publications No. 8023, revised 1996) and were approved by the ethics committee for animal research of the Shahid Beheshti University of Medical Sciences. All experiments were performed at the same time during the day to avoid circadian variations.

*Systemic drug injection*

**Figure 1:**
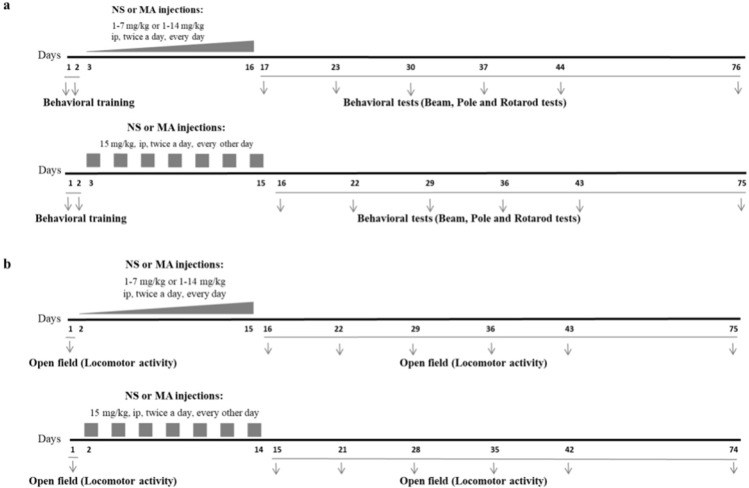
Experimental design. Three different MA regimens were administered. (a) Two sets of separate animals were used to evaluate motor performance parameters and (b) locomotor activity

Methamphetamine (MA) hydrochloride (synthesized and analyzed by Laboratory of Medicinal Chemistry, School of Pharmacy, Tehran University of Medical Sciences, Iran) was freshly dissolved in 0.9% saline solution before each administration. The rats received repeated escalating (1-7 mg/kg, or 1-14 mg/kg, IP, twice a day, for 14 consecutive days) or constant doses of MA (15 mg/kg, IP, twice a day, every other day for 7 injection days), at a volume of 1 mL/kg. Control group received injections of saline, IP, at the same volume twice a day, for 14 consecutive days. Escalating regimens were used in order to mimic human MA abuse, and constant regimen was used to induce neurotoxic effect of high doses of MA (21). In MA_1-7mg/kg _group injections began with 0.5 mg/kg in the first day, and gradually increased, 0.5 mg/kg per day. In MA_1-14mg/kg _group injections began with 1 mg/kg in the first day, and gradually increased, 1 mg/kg per day. In MA_15mg/kg _group, injections of MA were made every other day to reduce the mortality rate of animals in response to toxic doses. Injections were performed at 9:00 in the morning and 3:00 in the afternoon. Schematic illustration summarizing MA treatment regimens and later behavioral tests are provided in Figure 1.

*Body weight measurement*

The animals were weighed during injection period on days 1, 7, and 14 and during the two months of testing on days 1, 7, 14, 21, 28, and 60 as well. The body weights were compared between groups.

*Behavioral tests*

All rats were trained on the behavioral tests for two consecutive days before random assignment to MA or saline groups. At the 1^st^, 7^th^, 14^th^, 21^st^, 28^th^, and 60^th^ days after the last injections, the rats were subjected to behavioral assessments. In each testing day, beam test, pole test, and then rotarod test were performed subsequently, with 1 h intervals. Locomotor activity was recorded in naive conditions, given before the first injection in every group, and then on days 1, 7, 14, 21, 28, and 60 after last saline or MA injections. The observers were blind to groupsʹ assignment during the testing phase.

*Open field test*

Spontaneous general activity of animals in a novel environment was measured in a closed plexiglass box (40.64 cm × 40.64 cm) containing horizontal infrared sensors placed 2.5 cm above the arena floor (22). Each rat was placed in a chamber for a 5 min habituation period, and then horizontal activity was assessed for 20 min. Total number of beam breaks by each animal was recorded.

*Narrow beam test*

Narrow beam apparatus is a long wooden beam (100 cm in length, 4 cm wide and 3 cm depth) elevated 80 cm above the ground. A line is drawn 20 cm from the start end of beam, and a box is placed at the other end. During the training and testing sessions, the rats were placed entirely within the 20 cm starting zone facing its home cage and a stopwatch was started immediately upon release of the animal. The time to start walking (passing start zone) and total time on the beam were recorded. Before the beginning of injections, the animals received two consecutive days of training each consisting of five trials. Testing sessions consisting of five trials were performed as described above and the average of 5 trials per test session was considered as the final score (23).

**Figure 2:**
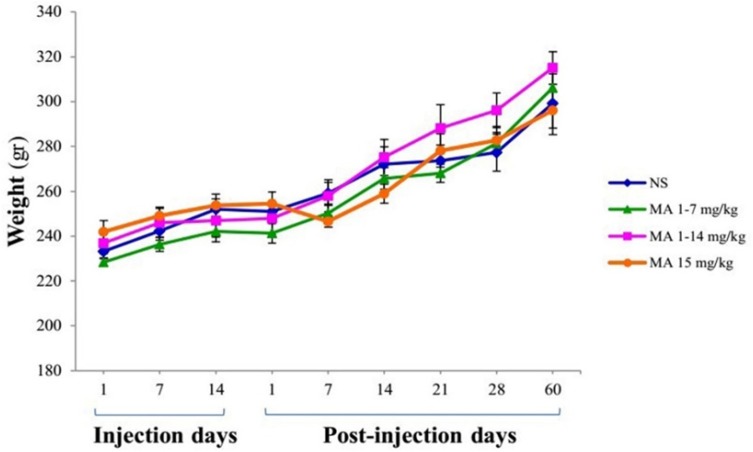
Body weight measurement. Rats received repeated escalating (1-7 mg/kg; n = 7, or 1-14 mg/kg; n = 8), or constant doses of MA (15 mg/kg; n = 9). Rats treated with normal saline (NS) served as controls (n = 7). Values are expressed as mean ± SEM. Repeated-measures ANOVA revealed no significant difference in body weight between groups

**Figure 3:**
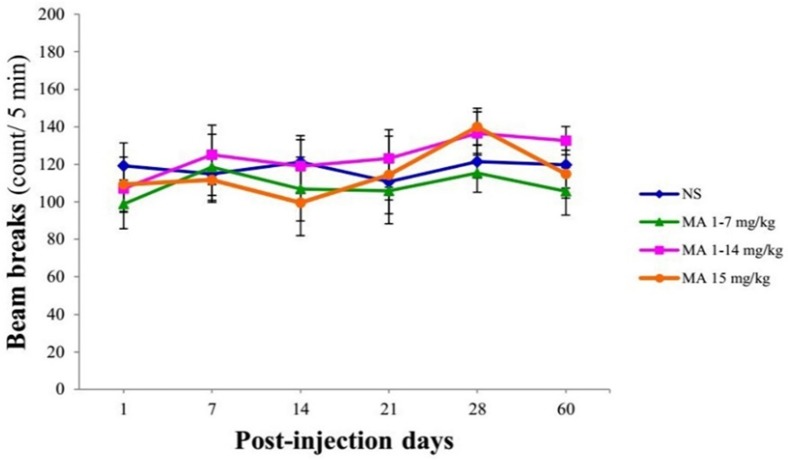
Locomotor activity using open-field test. Rats received repeated escalating (1-7 mg/kg; n = 7, or 1-14 mg/kg; n = 8), or constant doses of MA (15 mg/kg; n = 9). Rats treated with normal saline (NS) served as controls (n = 7). Values are expressed as mean ± SEM. Repeated-measures ANOVA revealed no significant difference in the number of beam breaks between groups

**Figure 4:**
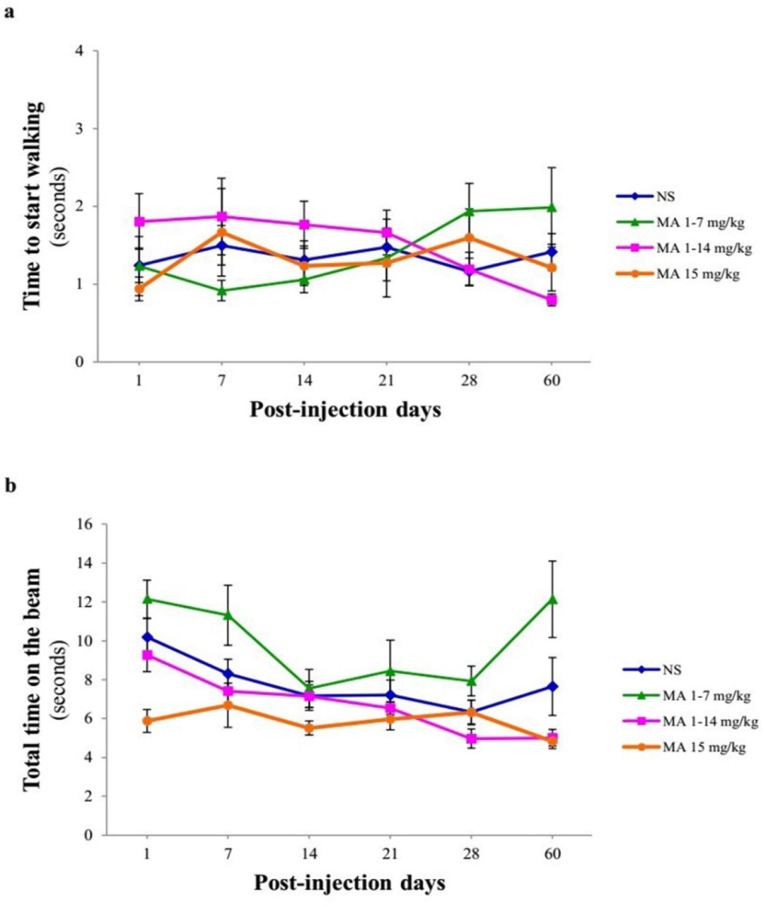
Motor performance and coordination using narrow beam test. Rats received repeated escalating (1-7 mg/kg; n = 12, or 1-14 mg/kg; n = 10), or constant doses of MA (15 mg/kg; n = 10). Rats treated with normal saline (NS) served as controls (n = 12). (a) Time to start walking and (b) total time on the beam were measured on 1, 7, 14, 21, 28 and 60 days after final injection. Values are expressed as mean ± SEM. Data analysis revealed that the time to start and total time on the beam were similar in normal saline- and MA-treated animals

**Figure 5:**
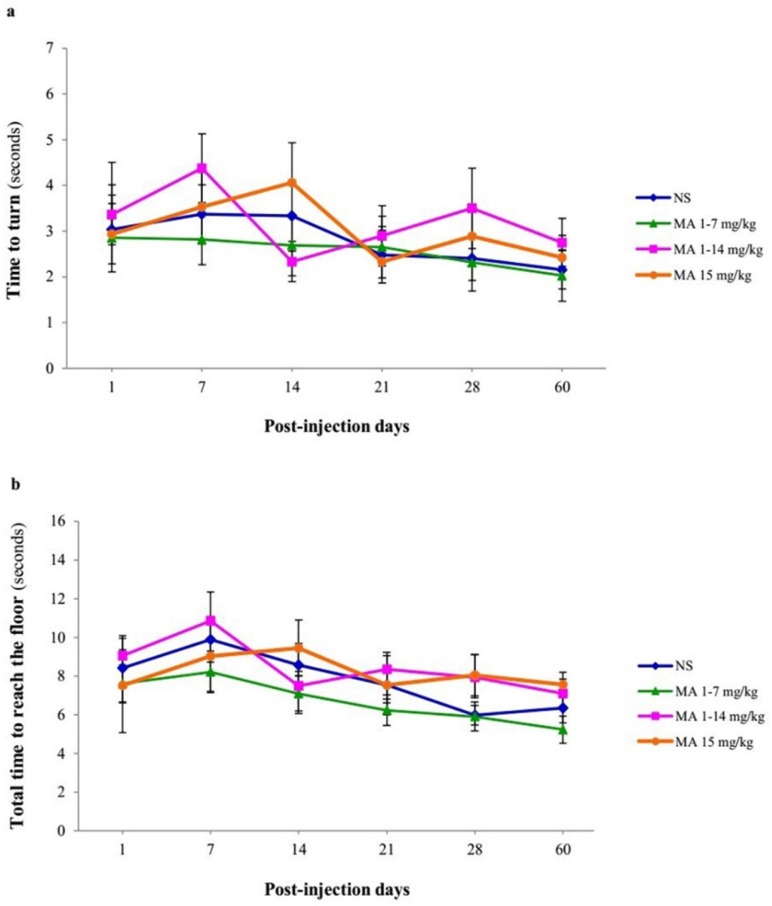
Motor performance and coordination using pole test. Rats received repeated escalating (1-7 mg/kg; n = 12, or 1-14 mg/kg; n = 10), or constant doses of MA (15 mg/kg; n = 10). Rats treated with normal saline (NS) served as controls (n = 12). (a) Time to turn and orient downward and (b) total time to traverse the pole (B) were measured on 1, 7, 14, 21, 28 and 60 days after final injection. Values are expressed as mean ± SEM. Repeated-measures ANOVA revealed no significant difference in time to turn and total time between groups

**Figure 6:**
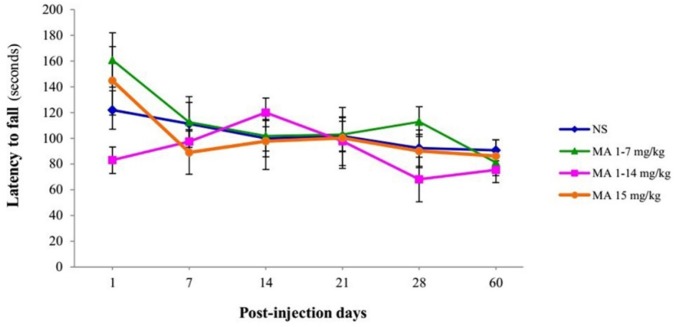
Assessment of latency to fall off the rotarod. Rats received repeated escalating (1-7 mg/kg; n = 12, or 1-14 mg/kg; n = 10), or constant doses of MA (15 mg/kg; n = 10). Rats treated with normal saline (NS) served as controls (n = 12). Latency time to fall off from rotarod with escalating speed were measured on and assessed behaviorally on 1, 7, 14, 21, 28 and 60 days after final injection. Values are expressed as mean ± SEM. Repeated-measures ANOVA revealed no significant difference between groups

*Pole test*

The rats were placed head-up on the top of the vertical metal pole (100 cm height, 2.5 cm diameter) and the “time to turn-around” and the “total time to reach the floor” were measured. Before the beginning of injections, the animals received two consecutive days of training each consisting of five trials. Testing sessions consisted of five trials with a maximum duration of 120 sec. The average of 5 trials per test session was considered as the final score (24).

*Rotarod test*

Motor performance was evaluated using a rotarod apparatus as previously described (25, 26). The rats were trained five times a day for 2 consecutive days until they could stay on the rotating drum for 300 sec. In the first training day, the rotarod speed was constant at 10 rpm, and in the second, it was accelerating from 5 to 20 rpm during 5 min. In the test sessions, the speed was increased from 5 to 40 rpm over 300 sec. Each rat performed 5 trials with 300 sec cut off in testing days and the average of 5 trials per test session was considered as the final score.

*Data analysis*

Behavioral results were statistically compared with repeated-measures ANOVA using 16^th^ version of SPSS. Analysis was followed by Dunnet post hoc test, for comparison between groups. All data were expressed as mean ± SEM and statistical significance was determined at *p* < 0.05.

## Results

*Body weight measurement*

The body weight average of rats was not significantly different between groups at the beginning or the end of the study (Figure 2). Repeated-measures ANOVA indicated no significant effect of treatment [F_ (3, 27)_ = 0.590; *p *= 0.628] and treatment × time interaction [F_ (5, 25)_ = 2.146; *p *= 0.122], but there is a significant effect of time [F_ (5, 25)_ = 159.932; *p *< 0.001].

*General locomotor activity*

Total numbers of beam break by the animals were compared between groups using repeated-measures ANOVA. Analysis indicated no significant effect of treatment × time interaction [F_ (5, 25)_ = 0.514, *p* = 0.928], treatment [F_ (3, 27)_ = 0.443; *p *= 0.725] and time [F_ (5, 25)_ = 1.817; *p *= 0.115] in the number of beam breaks between groups (Figure 3).

*Specified motor performance and motor coordination*

Narrow beam test measures skilled walking and requires basal ganglia influence on the pyramidal tract as well as the rubrospinal pathway. It has been shown that it detects nigrostriatal dopamine loss because it measures skilled walking. Furthermore, this test evaluates bradykinesia as an indicator of motor impairment in PD animal models (24). Repeated-measures ANOVA indicated no significant effect of treatment [F _(3, 40) _= 0.201, *p* = 0.895], time [F _(5, 38) _= 0.379, *p* = 0.859] and treatment × time interaction [F _(5, 38) _= 1.520, *p* = 0.113] in the time to start walking in MA-treated and control rats (Figure 4A). In case of total time on the beam, no significant effect of treatment F _(3, 40) _= 0.745, *p* = 0.41 was observed, while there was a significant effect of time [F _(5, 38) _= 9.066, *p* = 0.001] (Figure 4B).

The pole test was initially developed in 1985 by Ogawa as a measure of bradykinesia to be used in animal models of PD (27). 

The time to orient downward and total time to reach the floor was measured for each animal over 60 days after MA or saline discontinuation. Repeated-measures ANOVA revealed no significant differences in effect of treatment [F _(3, 40) _= 0.207, *p *= 0.891] and interaction of treatment × time [F _(5, 38) _= 1.317, *p *= 0.206] but significant effect of time [F _(5, 38) _= 5.279, *p *< 0.001] in time-to-turn (Figure 5A) and there were also no significant differences in effect of treatment [F _(3, 40) _= 0.841, *p *= 0.480] and interaction of treatment × time [F _(5, 38) _= 1.112, *p *= 0.356], but significant effect of time [F _(5, 38) _= 3.826, *p *= 0.008], in total time to reach the floor (Figure 5B) between groups. In other words, MA-treated animals performed pole test similar to control animals.

In the case of motor coordination, repeated-measures ANOVA revealed that there is no significant difference between groups effect of treatment [F _(3, 40) _= 0.632, *p* = 0.600] in latencies to fall off from the rotating rod (Figure 6).

## Discussion

Our results revealed that MA at the applied regimens has no effects on motor performance, coordination, and locomotor activity in rats. Reviewing previous studies evaluating the effect of MA administration on motor activities in animals, the controversial and conflicting results can be attributed to several factors such as dose of MA, exposure duration (acute or repeated), dosing intervals, and different type or strain of animals used (21, 28 and 29).

In the case of MA doses, studies have shown that acute moderate and high doses of MA applied in one or more injections in single day trials cause motor impairments in rodents, but these effects appear differently. For example, a single high dose of MA (30 mg/kg) in mice decreases locomotor activity in open field test just 2.5 days following injection, and results in motor coordination deficits in rotarod test after 12 weeks (30). In another study, a significant deficit in balance beam performance (2- to 3-fold increase in footfalls) was seen one week after MA at the dose of 12.5 mg/kg injected for 4 times in a day (31). However, motor deficits produced by acute moderate to high doses persist for a short time which may be due to the recovery of dopaminergic terminals. In this regard, Liu, Shi *et al.* and Ares-Santos *et al.* have shown that multiple administration of MA in a single day significantly shortens the time on the rotarod at days 1 and 3, indicating impaired motor balance. This impaired fall latency recovers gradually at day 7, which is consistent with recovery of dopamine content in the striatum at the same time (32, 33).

In contrast to studies mentioned above, which used constant MA doses in a single day, we administered MA in two escalating regimens beginning with low doses. It has been shown that chronic escalating doses of MA produce preconditioning effect which result in complete protection against dopamine depletion in the striatum (1). Repeated injections of nontoxic MA doses can increase glial-derived neurotrophic factor (GDNF) (6), small heat shock proteins (HspB1 and HspB2), brain-derived neurotrophic factor (BDNF), and hem oxygenase-1 (Hmox-1), in the rat striatum. These responses of intrinsic striatal cells develop a certain degree of tolerance to MA-induced dopaminergic damages in the animals (34). Consistent with animal studies, prior exposure to sub-toxic concentrations of MA protects dopaminergic cells against 6-hydroxy dopamine toxicity in culture, whereas higher concentrations of MA exacerbate it. MA up regulates the pro-survival protein Bcl-2 in these cells and therefore results in a decrease in their vulnerability to subsequent oxidative stress (35). Considering these points, no significant motor deficits in our two escalating regimens can be attributed to a preconditioning effect of first exposure to lower doses of MA.

Repeated administrations of MA induces locomotor sensitization and results in changes in locomotor activity of animals which is characterized by a progressive increase in locomotor response after each injection (36). However, our behavioral assessment was conducted when the animals were not still in the sensitized state. Thus, the current findings do not contradict the literature data, especially because the behavioral evaluations were not carried out under MA influence, but during its abstinence period. In accordance with us, in one recent study MA was administered in an escalating dose regimen from 0.2 to 6.0 mg/kg, during a 16-day period in pre-adolescent rats. Locomotor activity assessment 25 days after drug discontinuation indicated that MA didn’t produce any significant differences in locomotion. Thus, MA-induced decreased locomotor activity seems to be an immediate response due to altered central monoamine levels, and is not long-lasting after discontinuations (37). Iijima *et al.* have demonstrated that repeated doses of MA (5 mg/kg, every day for 5 days) didn’t decrease locomotor activity of rats measured at 2 days following withdrawal (38). In addition, in a recent study, locomotor activity of rats has been assessed at day 17 after MA (4 mg/kg for 8 days) discontinuation using Y-maze and no significant changes in general activity of animals in MA-treated group has been reported (39), which is consistent with our findings.

It has been shown that high doses of MA (20 mg/kg, every day for 5 days) induces significant coordination deficit in the rotarod test and open field test on days 6, 10, and 14 after final injection (40). It is possible that 24-hour intervals between injections in MA_15mg/kg _group in our study, provide an opportunity for activation of compensatory mechanisms, and so no significant behavioral deficits in this group can be observed despite the high doses prescribed. In some human MA-abusers, enlargement of the striatum (putamen and globus-pallidus) has been shown which may represent a compensatory response to maintain function and motor performance similar to controls. Possible mechanisms underlying striatal enlargement include glial activation and inflammatory changes associated with MA-induced injury (41). Moreover, it has been shown that MA exposure induces cell proliferation in the striatum accompanied by enlarged striatal volume (up to 50% larger than controls) in mice (42). Some of these new generated cells, derived from dormant striatal progenitors, differentiate into neurons at later time point’s post-MA. Dormant progenitors represent a potential source of new cells to replace the loss of some striatal neurons in chronic MA abuse (30).

The clinical features of PD develop once about 50% of dopaminergic neurons lost in human (43). In animal model of PD motor impairment appears only after an 80% loss of striatal dopamine and 30–60% loss of nigral dopamine neurons, and persistent 25% loss of nigral neurons produces no motor symptoms (43, 44), indicating that MA could destroy many dopaminergic neurons with no parkinsonian symptomatology (45), and therefore MA-induced toxicity appear only at the cellular and molecular levels.

## Conclusion

There is a controversy about the effects of MA on PD-related motor behavior in animal models. Possibly MA dose, exposure duration, and dosing intervals are important factors in characterizing MA effects on dopamine-related behaviors. A variety of several compensatory mechanisms such as increase in neurotrophic factors, preconditioning effect, neurogenesis in striatum, and enlarged basal ganglia structures following MA exposure, may attenuate MA-induced dopaminergic toxicity, to be far from manifested at motor behavior.
